# Quiet Quitting in Nursing: The Role of Psychological Well‐Being, Person‐Group Fit, and Generational Differences

**DOI:** 10.1155/jonm/2836032

**Published:** 2026-09-18

**Authors:** Shu-Chuan Jennifer Yeh, Hsueh-Chih Chou, Ying-Ying Lo

**Affiliations:** ^1^ Institute of Health Care Management & Department of Business Management, National Sun Yat-Sen University, Kaohsiung, Taiwan, nsysu.edu.tw; ^2^ Department of Healthcare Administration and Medical Informatics, Kaohsiung Medical University, Kaohsiung, Taiwan, kmu.edu.tw; ^3^ Department of Nursing, Kaohsiung Veterans General Hospital, Kaohsiung, Taiwan, vghks.gov.tw; ^4^ Shu-Zen Junior College of Medicine and Management, Kaohsiung, Taiwan, szmc.edu.tw; ^5^ Department of Healthcare Administration, I-Shou University, Kaohsiung, Taiwan, isu.edu.tw

**Keywords:** generation, mediation, moderated mediation, person-group fit, psychological well-being, quiet quitting

## Abstract

Quiet quitting, where employees restrict their contributions to the minimum required by their roles, has become an urgent challenge in today’s workplaces. This study examined whether person‐group fit mediates the relationship between psychological well‐being and quiet quitting among nurses and whether this indirect relationship varies across generations. A three‐wave, time‐lagged survey of 997 registered nurses representing a multigenerational workforce was conducted. Mediation and moderated mediation analyses were used to test the proposed relationships and explore potential generational differences. Results showed that psychological well‐being was negatively associated with quiet quitting, both directly and indirectly through person‐group fit. Although the indirect effects showed some variation across generations, the index of moderated mediation was not statistically significant, indicating that generational differences in the strength of the indirect pathway were not supported. Thus, person‐group fit may represent one mechanism linking psychological well‐being to quiet quitting, while the extent to which this mechanism varies across generations remains unclear. The observed generational patterns should therefore be interpreted as exploratory and may require further investigation considering factors such as career stage, workplace priorities, and contextual influences. This study highlights the potential value of engagement strategies that support psychological well‐being and strengthen person‐group fit within multigenerational nursing teams. Given the modest effect sizes, nonsignificant moderation findings, and study design limitations, the findings should be interpreted with caution. Future research should further examine how career stage, workplace values, and contextual factors influence the relationship between psychological well‐being, person‐group fit, and quiet quitting.

## 1. Introduction

Quiet quitting (QQ)—defined as employees intentionally restricting their contributions to formal job requirements while remaining employed—has emerged as a subtle yet consequential form of behavioral withdrawal in contemporary workplaces [1]. Rather than reflecting complete psychological disengagement, QQ involves the withdrawal of discretionary or extra‐role effort, often in response to dissatisfaction with organizational support, ineffective leadership, excessive workloads, and perceived unfairness [2–4]. Importantly, QQ is conceptualized here as a within‐role behavioral regulation strategy rather than a general syndrome of attitudinal or psychological disengagement. From a Social Exchange Theory (SET) perspective, QQ reflects a reciprocity‐based behavioral adjustment in which employees reduce discretionary effort when perceived organizational inducements are insufficient to justify continued extra‐role contribution. Such conditions may signal a breach of the psychological contract or an imbalance in work exchanges. Importantly, psychological contract breach and effort‐reward imbalance are conceptualized as antecedent conditions rather than defining features of QQ. Over time, these adverse work environments may erode trust, collaboration, and engagement [5], thereby reducing employees’ willingness to contribute beyond formal role requirements. This reflects a gradual depletion of relational and psychological resources needed for sustained discretionary effort [6, 7].

Despite its rapid diffusion in managerial discourse, QQ remains conceptually ambiguous because it overlaps with several established constructs related to reduced work involvement and employee withdrawal. A key theoretical question is whether QQ represents a distinct behavioral phenomenon or merely a contemporary label for existing concepts, including low work engagement, reduced organizational citizenship behavior (OCB), work withdrawal, and turnover intention. Establishing its conceptual distinctiveness is therefore essential for positioning QQ within the broader nomological network of employee work behaviors. To address this issue, we developed a nomological network (Appendix A) that systematically compares QQ with these theoretically related constructs, highlighting their defining characteristics as well as key similarities and distinctions.

Conceptually, QQ occupies a distinct position among withdrawal‐related constructs by reflecting a deliberate recalibration of effort within formal role boundaries rather than a generalized decline in work attachment or an intention to leave the organization. Unlike low work engagement, which reflects diminished vigor, dedication, and absorption, QQ does not necessarily imply reduced psychological involvement; employees may remain committed to their work while intentionally limiting effort beyond contractual expectations. It is also distinct from OCB. Whereas OCB refers to voluntary behaviors that exceed formal job requirements, QQ reflects the intentional withholding of discretionary, extra‐role contributions while maintaining expected in‐role performance. Similarly, QQ differs from broader forms of work withdrawal, which encompass behavioral and cognitive disengagement (e.g., absenteeism, lateness, presenteeism, and withdrawal cognitions), because employees continue to fulfill their formal responsibilities despite reducing discretionary effort. Finally, unlike turnover intention, which reflects a conscious intention to leave the organization, QQ represents an adjustment in effort investment while remaining employed. Taken together, these distinctions suggest that QQ is best understood as a unique pattern of behavioral adaptation characterized by reduced discretionary contribution rather than diminished psychological engagement, organizational withdrawal, or anticipated exit.

This pattern is particularly salient in nursing contexts. Empirical studies consistently indicate that poor work environments, low leadership support, workplace bullying, and insufficient personal resilience are associated with higher levels of QQ and reduced work engagement among nurses [8–12]. Cross‐sectional evidence further suggests that nurses exhibit a higher prevalence of QQ compared with other healthcare professionals, with burnout and job dissatisfaction emerging as key antecedents [8, 12]. Moreover, QQ among nurses has been shown to significantly increase turnover intention, directly linking this form of behavioral withdrawal to persistent workforce retention challenges in healthcare settings [13]. Within the SET framework, these findings suggest that repeated exposure to resource‐depleting work environments disrupts perceived exchange reciprocity, thereby increasing the likelihood of effort withdrawal as a self‐protective response.

While QQ can serve as a coping mechanism to preserve work‐life balance and prevent burnout [14], it may also weaken team cohesion, reduce productivity, and elevate turnover risks, particularly among high‐performing staff [1]. This dual nature challenges the stereotype of quiet quitters as simply unproductive and highlights the importance of understanding QQ as a multifaceted, context‐dependent phenomenon. Accordingly, within a reciprocity‐based exchange framework, QQ may be understood as a protective withdrawal of discretionary investment in response to perceived imbalance in workplace exchanges.

This study is grounded in SET, which suggests that employees reciprocate supportive and fair workplace relationships through continued engagement and discretionary effort [15]. Within this framework, psychological well‐being (PWB) is conceptualized as a relationally embedded resource that facilitates positive social exchange processes within work groups, thereby enhancing perceived person‐group fit (PG fit), whereas perceived imbalance in workplace exchanges may contribute to QQ behaviors. Accordingly, SET provides the theoretical basis linking PWB, PG fit, and QQ in nursing workplaces.

Research consistently indicates that poor PG fit undermines employee well‐being and increases disengagement risk [16]. PG fit reflects the degree of alignment between employees and their work groups in terms of values, attitudes, and interpersonal compatibility. From a social exchange perspective, PWB may foster higher‐quality interpersonal exchanges, including trust‐building, helping behaviors, and emotional expressiveness, which in turn enhance perceived PG fit. Higher PWB may facilitate positive social exchanges and stronger group integration, whereas value misalignment may contribute to discomfort, withdrawal, and reduced discretionary effort [17, 18]. Accordingly, PG fit may function as a key mechanism linking PWB to QQ.

In addition, examining QQ within an East Asian context is particularly meaningful. Healthcare organizations in East Asian societies such as Taiwan are characterized by relatively high levels of collectivism and power distance [19], where interpersonal harmony, group belonging, and hierarchical relationships are strongly emphasized. Accordingly, PG fit may be particularly salient in such contexts, as alignment with group norms and values is likely to exert a stronger influence on behavior in collectivist cultures compared to more individualistic settings. In collectivist healthcare environments, such relational exchange processes may be particularly influential in shaping employees’ perceptions of reciprocity and group alignment.

Within nursing teams, where work processes are highly interdependent and value‐driven, misalignment between individual and team values has been linked to emotional exhaustion, weakened collaboration, and disengagement from work roles [20]. These dynamics may be particularly salient among Generation Z employees, who are often characterized as more sensitive to value incongruence and perceived misalignment with group norms, increasing their vulnerability to psychological detachment and disengagement [21, 22].

Generational differences are increasingly important in healthcare workforce management, as staffing shortages and turnover threaten organizational stability and patient outcomes. Prior research indicates that Millennials and Generation Z report lower work centrality, intrinsic motivation, and organizational commitment than older cohorts, alongside stronger preferences for autonomy and work‐life balance [21, 23]. Drawing on generational theory, individuals sharing formative historical and sociocultural conditions may develop similar orientations toward work and organizational life [24, 25]. However, contemporary perspectives further emphasize that generational differences should not be interpreted as inherent characteristics of birth cohorts but rather as socially and structurally shaped responses to changing workplace conditions and sociocultural contexts [26].

Accordingly, younger cohorts may enter the workforce with expectations shaped by contemporary sociocultural and institutional contexts, including greater emphasis on PWB, flexibility, and supportive, growth‐oriented environments [21, 23, 27, 28]. These expectations may be particularly salient in nursing contexts characterized by high emotional demands, strong interdependence, and hierarchical structures. Because nursing practice requires continuous communication, emotional regulation, and reliance on colleagues for coordination and support, nurses’ PWB may influence how they perceive their compatibility, belonging, and connection within their work groups. Therefore, PWB may contribute to stronger PG fit by facilitating positive perceptions of interpersonal relationships and shared workplace experiences. However, this association may vary across generational cohorts because different generations may place different levels of importance on relational support, psychological safety, and workplace connectedness.

Younger cohort nurses may be more likely to base their perceptions of compatibility with their work groups on their PWB because they generally place greater value on supportive, meaningful, and psychologically healthy work environments [21, 23, 27, 28]. Consequently, greater PWB may enhance their perceptions of interpersonal compatibility, belonging, and connectedness within their work groups, resulting in a stronger positive association between PWB and PG fit. In contrast, Generation X nurses, whose professional identities were shaped within more traditional organizational contexts emphasizing autonomy, professional commitment, and adaptation to workplace demands, may rely less on interpersonal alignment when evaluating their relationship with their work groups. Consequently, the relationship between PWB and PG fit may be weaker among older cohorts.

Generational differences may also shape how PG fit influences QQ behaviors. In nursing environments, where teamwork and interpersonal coordination are essential for maintaining patient care quality and managing occupational stress, strong PG fit may function as an important psychological and social resource. For younger cohort nurses, unmet expectations regarding supportive and meaningful workplace relationships may represent a stronger discrepancy between desired and actual workplace experiences, thereby increasing the likelihood of psychological withdrawal and QQ. In contrast, older cohorts may demonstrate greater tolerance of work strain and interpersonal misalignment because of their earlier professional socialization. This greater tolerance may reduce the extent to which poor PG fit translates into withdrawal behaviors among older nurses.

Moreover, the postpandemic healthcare environment, marked by increased workloads, burnout, and staffing shortages, may further amplify generational differences in responses to workplace stressors and group dynamics. Following COVID‐19, heightened awareness of PWB, workplace support, and team functioning may have increased the importance of relational experiences in determining nurses’ engagement and retention. Under these conditions, generationally shaped values may influence how nurses evaluate their workplace relationships and respond to experiences of group misalignment. In such contexts, generationally shaped values may heighten sensitivity to misalignment with work groups, weakening PG fit and increasing the likelihood of QQ when PWB is compromised.

Accordingly, this study hypothesizes that PG fit mediates the relationship between PWB and QQ and that this indirect effect is moderated by generational cohort (Generation Z, Y, and X), such that the mediation varies across cohorts. Specifically, generational cohort may influence both the extent to which PWB translates into perceptions of PG fit and the extent to which PG fit affects withdrawal‐related behaviors. Recognizing these dynamics is essential for understanding disengagement and informing targeted engagement and retention strategies in a multigenerational nursing workforce.

Although “generation” is conceptually complex and shaped by shared historical, cultural, and institutional experiences [24, 25, 29], empirical research commonly operationalizes it using birth cohorts [29]. Consistent with Mannheim’s theory of generational formation, individuals within the same cohort may share exposure to similar contexts that shape their values and workplace expectations [24, 25]. In this study, nurses were classified into Generation Z, Y, and X based on birth year. However, generational categories should not be interpreted as fixed or homogeneous, as substantial within‐cohort heterogeneity exists. Observed differences may also reflect age, career stage, or period effects rather than purely cohort‐based influences [30]. Nevertheless, cohort‐based classification remains a widely used and pragmatic heuristic in organizational research for examining broad patterns in work‐related attitudes and behaviors.

The purpose of this study is to examine the relationship between nurses’ PWB and QQ, to test the mediating role of PG fit, and to investigate whether generational cohort moderates this indirect effect. Recent research has identified workplace bullying, work environment, and engaging leadership as important factors associated with QQ among nurses [8–10], but it is not known about the psychological and relational processes linking nurses’ well‐being to QQ. Building on this emerging literature, the present study extends prior research in three ways. First, it extends the focus beyond adverse workplace conditions and leadership factors by examining PWB as an individual‐level antecedent of QQ, thereby broadening the range of factors considered in explaining this behavior. Second, the study advances understanding of how PWB relates to QQ by identifying PG fit as a potential mediating mechanism linking well‐being to QQ, highlighting the role of team‐level value and relational alignment. Third, by testing whether this indirect association varies across generational cohorts, the study examines the extent to which the relationships among PWB, PG fit, and QQ may differ across Generation Z, Y, and X nurses. Together, these contributions extend the emerging nursing literature on QQ by integrating individual well‐being, group‐level fit, and generational context within a single theoretical model.

## 2. Methods

### 2.1. Study Design and Participants

This study employed a three‐wave survey design involving registered nurses from a large medical center in Taiwan. The selection of the time lags was guided by methodological recommendations in occupational health psychology and longitudinal research. Prior studies highlight that appropriate time lags should correspond to the expected temporal dynamics of the constructs under investigation, as overly short or overly long intervals may obscure longitudinal effects [31, 32]. As there is no universal optimal interval, lag selection should be theory‐driven and construct‐specific [33].

Accordingly, intervals of approximately one to 2 months between waves were adopted to capture short‐ to medium‐term changes in PWB, PG fit, and QQ behavior. This time frame is appropriate given evidence that work‐related attitudes, perceived fit, and withdrawal behaviors may fluctuate over relatively short periods, while PWB may change more gradually but still shows measurable variation over months in response to work experiences. These constructs are therefore conceptualized as dynamic work‐related states rather than stable traits. Nevertheless, these intervals are more suitable for detecting proximal changes in work‐related states and may not capture slower developmental processes or cumulative effects that emerge over extended periods.

To reduce potential common method bias and strengthen temporal ordering among variables, data on key constructs were collected at different time points across the three waves. Eligibility criteria included registered nurses who had been employed at the hospital for more than 3 months at the time of data collection. Data were collected using structured paper‐and‐pencil questionnaires administered by three trained research assistants. Prior to data collection, the principal investigator provided standardized training to ensure consistency in survey administration and adherence to the study protocol. Participants completed the questionnaire within approximately 10–15 min and returned it upon completion.

In Wave 1 (December 2024), 1200 questionnaires were distributed, and 1092 were returned (response rate = 91.0%), assessing PWB and demographic characteristics. In Wave 2 (January 2025), the 1092 Wave 1 respondents were invited to participate, yielding 1038 valid responses measuring person–group fit. In Wave 3 (March 2025), QQ behavior was assessed among the 1038 Wave 2 respondents, with 997 completed responses obtained. The final sample comprised 997 nurses who completed all three waves, representing an overall retention rate of 83.1%.

### 2.2. Measures

#### 2.2.1. QQ

QQ behavior was measured using an eight‐item instrument adapted from Anand et al., rated on a seven‐point Likert scale, with higher scores indicating greater levels of QQ [34].

To ensure content validity in the healthcare context, an expert review procedure was conducted involving five experts from nursing (*n* = 2), occupational psychology (*n* = 1), organizational behavior (*n* = 1), and healthcare management (*n* = 1). Each expert evaluated item relevance using a 4‐point Likert scale (1 = not relevant, 4 = highly relevant). Content validity was assessed using the item‐level content validity index (I‐CVI) and scale‐level content validity index (S‐CVI), with an I‐CVI threshold of ≥ 0.78 serving as the criterion for item retention. Items failing to meet this threshold were considered for removal based on expert consensus regarding conceptual relevance and contextual appropriateness.

Seven items achieved perfect expert agreement (I‐CVI = 1.00), whereas one item (“I often avoid working more hours if there is no additional pay”) demonstrated insufficient relevance for the clinical context (I‐CVI = 0.00)^1^. Experts indicated that the item did not adequately reflect nursing practice, where decisions regarding overtime are often influenced by patient care responsibilities and professional obligations rather than solely by financial considerations. Consequently, the item was removed. Following item deletion, the content validity of the revised seven‐item scale was re‐evaluated and demonstrated acceptable overall content validity (S‐CVI/Ave = 0.875) (Appendix B).

The revised instrument included items such as “I often arrive late and leave early from work.” The seven‐item scale demonstrated good internal consistency in the current sample (Cronbach’s *α* = 0.852). Confirmatory factor analyses (CFAs) further supported the construct validity of the adapted instrument, and measurement invariance across generational groups was subsequently examined (see Section 3.1).

#### 2.2.2. PWB

PWB was assessed using the 14‐item Warwick–Edinburgh Mental Well‐being Scale [35], which has been validated in Chinese populations [36]. Participants rated how often each statement described their experiences over the past 2 weeks on a 5‐point scale (1 = never and 5 = all of the time). A sample item is “I am feeling optimistic about the future.” The scale demonstrated excellent internal consistency in this study (*α* = 0.952).

#### 2.2.3. PG Fit

PG fit was assessed using six items, including three items measuring person–organization fit and three items measuring demands–abilities fit [37]. Responses were recorded on a 7‐point Likert scale (1 = strongly disagree and 7 = strongly agree). Sample items include: “My personal values match my team’s values and culture” and “Our team members’ abilities and training are a good fit with the requirements of the team tasks.” All items were translated into Chinese using Brislin’s forward–backward translation procedure, with discrepancies resolved through discussion and pilot testing to ensure clarity and cultural appropriateness [38]. Prior research has demonstrated high reliability for both subscales (*α* = 0.90) [16]. In the present study, the combined scale also showed good internal consistency in the current sample (*α* = 0.895).

#### 2.2.4. Generations

Participants were classified into generational cohorts based on birth year—Generation X (1965–1979), Generation Y (1980–1995), and Generation Z (born after 1995) [39]—as a heuristic proxy for generational identity.

#### 2.2.5. Control Variables

We included tenure and marital status as control variables due to their potential influence on employee behavior and attitudes. Tenure was controlled because it can affect employees’ entitlements to paid leave and is often associated with higher levels of job‐relevant knowledge and experience [40]. Employees with longer tenure are likely to have developed greater expertise [41], which is a strong predictor of job performance. Marital status was also included, as prior research suggests it may be associated with key workplace outcomes such as turnover, absenteeism, and organizational commitment [42].

### 2.3. Statistical Analysis

To address potential concerns regarding common method bias, we used process control using a three‐wave survey design and examined Harman’s single‐factor test. An unrotated principal component factor analysis revealed that the first factor accounted for 23.17% of the total variance, indicating that common method bias is not a major concern in this study.

Descriptive and correlation statistics were performed using IBM SPSS version 28.0 [43]. Second, the mediation effect was examined using Hayes’s Process macro (Model 4). Third, we employed PROCESS Model 59 to test a moderated mediation model in which Generation moderates the effect of X on Mediator (path a), the effect of Mediator on Y (path b), and the direct effect of X on Y (path c’). Consequently, the indirect effect of X on Y through Mediator is also conditionally moderated by Generation. This approach allowed us to examine whether the mediation process varied across generational cohorts. We used the bootstrapping method to determine whether the significance of the mediation effects is according to a moderator’s value [44].

In addition, measurement invariance across generational groups (Gen X, Gen Y, and Gen Z) was assessed using multigroup CFA. Configural invariance was first tested to examine whether the factor structure was consistent across groups, followed by metric invariance, in which factor loadings were constrained to be equal. Model comparisons were evaluated using changes in *χ* ^2^, CFI, and RMSEA.

### 2.4. Ethical Considerations

This present study was approved by the institutional review board of Kaohsiung Veterans General Hospital (KSVGH24‐CT1‐03). In addition, written informed consent was obtained from all participants.

## 3. Results

### 3.1. Measurement Model and Invariance Testing

We conducted CFA to evaluate the construct validity of the adapted QQ scale. The results indicated acceptable model fit for the overall sample (*χ* ^2^ (14) = 191.96, CFI = 0.938, TLI = 0.906, RMSEA = 0.082, and SRMR = 0.048). Across generational groups (Gen X, Gen Y, and Gen Z), the model also demonstrated generally consistent fit, with CFI values ranging from 0.938 to 0.945, TLI from 0.908 to 0.918, RMSEA from 0.076 to 0.088, and SRMR values consistently below 0.05.

All standardized factor loadings exceeded 0.60. Composite reliability (CR) values ranged from 0.835 to 0.884, and average variance extracted (AVE) values ranged from 0.533 to 0.630, supporting satisfactory convergent validity and internal consistency (Table S1).

Measurement invariance across generational groups was assessed using multigroup CFA. Configural invariance was supported (CFI = 0.929, RMSEA = 0.073), indicating a consistent factor structure across groups. Metric invariance was also supported, as constraining factor loadings did not significantly worsen model fit (Δ*χ* ^2^ = 12.893, Δdf = 12, *p* = 0.377; ΔCFI = 0.003; ΔRMSEA = 0.007), thereby indicating measurement equivalence across generational cohorts (Table S2).

### 3.2. Descriptive and Univariate Analyses

Table 1 summarizes participants’ demographics and characteristics. Nurses averaged 36 years of age (SD = 11.2), 12.3 years of experience (SD = 10.3), and 6.5 h of sleep daily (SD = 1.1). Most were female (94.3%), and 39.3% were married. QQ was negatively correlated with PWB (*r* = −0.38, *p* < 0.01) and PG fit (*r* = −0.48, *p* < 0.01), while well‐being was positively related to PG fit (*r* = 0.42, *p* < 0.01), with correlations in the moderate range (Table 2).

**TABLE 1:** Demographics of clinical registered nurses (*N* = 977).

Variables	Frequency (mean)	Percent (%) (s.d)
Age	(36)	(11.2)
Generations		
Z	391	40.2
Y	390	40.1
X	191	19.7
Work experience		
Total years of work experience	(12.32)	(10.26)
Years of experience at current unit	(6.97)	(7.75)
Daily time spent online	(5.08)	(3.05)
Daily sleep duration	(6.51)	(1.11)
Gender		
Male	55	5.66
Female	917	94.34
Marital status		
Married	380	39.26
Not married	588	60.74

**TABLE 2:** The means, standard deviation, and correlations among study variables.

	Mean	SD	1	2	3	4	5	6
1. Age	36	11.2	—					
2. Tenure	12.32	10.26	0.962^∗∗^	—				
3. Marital status	0.39	0.49	0.574^∗∗^	0.564^∗∗^	—			
4. PW	47.11	8.86	0.120^∗∗^	0.112^∗∗^	0.115^∗∗^	0.952		
5. PG fit	28.22	5.70	0.076^∗^	0.076^∗^	0.063	0.422^∗∗^	0.895	
6. Quiet quitting	20.33	7.89	−0.084^∗∗^	−0.080^∗^	−0.075^∗^	−0.379^∗∗^	−0.475^∗∗^	0.852

*Note:* PW = psychological well‐being, PG fit = person‐group fit; The diagonal values on the diagonal represent Cronbach’s alpha coefficients.

^∗^ *p* < 0.05.

^∗∗^ *p* < 0.01.

^∗∗∗^ *p* < 0.001.

Among participants, 40.2% were Generation Z, 40.1% Generation Y, and 19.7% Generation X. Generational differences were found in tenure, well‐being, and QQ. Generation Y reported the highest QQ scores (M = 21.1, SD = 8.0), followed by Generation Z (M = 20.5, SD = 7.7) and Generation X (M = 18.3, SD = 7.7), with younger generations scoring significantly higher than Generation X, although the magnitude of these differences was modest. Conversely, Generation X exhibited the highest PWB (M = 46.5, SD = 8.8), differing significantly from Generation Z. Although Generation Y had the highest mean PG fit, followed by Generation X and Generation Z, these differences were not statistically significant (Table 3).

**TABLE 3:** Generation differences (*N* = 972).

Variables	GEN Z (*n* = 391)		GEN Y (*n* = 390)		Gen X (*n* = 191)		*F*	*p*	Scheffe
	Mean	SD	Mean	SD	Mean	SD			
Tenure	3.19	3.80	13.66	4.93	28.33	5.26	1907.617	< 0.001	Z < Y < Z
Psychological well‐being	46.49	8.82	47.04	8.75	48.54	8.88	3.492	0.031	Z < X
Person‐group fit	27.83	5.35	28.55	5.99	28.33	5.71	1.602	0.202	NA
Quiet quitting	20.45	7.69	21.09	8.01	18.32	7.65	8.114	< 0.001	Z > X; Y > X

### 3.3. The Mediating Effect of PG Fit

PG fit was examined as a mediator between PWB and QQ using Hayes’ (2012) PROCESS macro (Model 4), with all variables standardized (Z‐scores). PWB was negatively associated with QQ (c′ = −0.218, 95% CI [−0.299, −0.139]) and positively associated with PG fit (a = 0.237, 95% CI [0.180, 0.294]). PG fit was negatively associated with QQ (b = −0.371, 95% CI [‐0.528, −0.217]).

**FIGURE 1:**
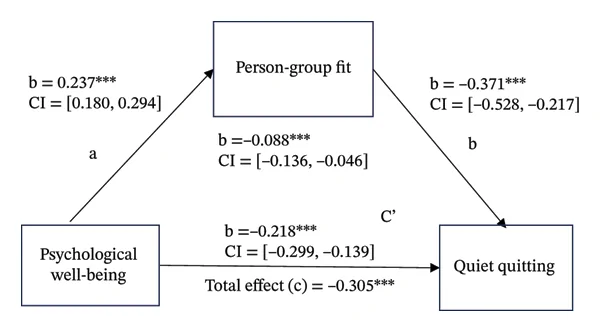
Person‐group fit served as a mediating variable in the relationship between psychological well‐being and quiet quitting. Notes: (1) path a represents the effect of psychological well‐being on person‐group fit; path b represents the effect of person‐group fit on quiet quitting; and path c′ represents the direct effect of psychological well‐being on quiet quitting after accounting for the mediator. (2) The figure presents standardized path coefficients. Total effects and their 95% confidence intervals are highlighted in bold. The direct effect and its 95% confidence interval are shown above the arrows, while the indirect effect and its 95% confidence interval—through the mediating variable (person‐group fit). (3) The analysis was based on 5000 bootstrap samples using 95% bias‐corrected confidence intervals, with tenure and marital status controlled. (4) ^∗^ *p* < 0.05; ^∗∗^ *p* < 0.01; ^∗∗^ *p* < 0.001.

The indirect effect was significant (−0.088, 95% CI [−0.136, −0.046]), indicating partial mediation. The total effect was −0.305 (95% CI [−0.358, −0.252]). Although the indirect effect was statistically significant, its magnitude was relatively small, suggesting that PG fit represents one contributing mechanism linking PWB and QQ rather than a dominant explanatory pathway. Therefore, the findings should be interpreted in terms of statistical association rather than implying a large practical effect. These findings support the partial mediating role of PG fit (Table 4; Figure 1).

**TABLE 4:** The mediation model of person‐group fit.

Variables	Coefficient	Boot LLCI	Boot ULCI	Significance
*Outcome Variable: PG Fit*				
Constant	16.996	14.413	19.528	yes
PW	0.237	0.180	0.294	yes
Tenure	−0.005	−0.047	0.035	No
Married	0.416	−0.458	1.311	No

**R-squared**	**F value**	**p** **value**		
0.138	49.939	< 0.001		

*Outcome Variable: Quiet Quitting*				
Constant	41.784	38.734	44.751	yes
PW	−0.218	−0.299	−0.139	yes
PG fit	−0.371	−0.528	−0.217	yes
Tenure	−0.067	−0.116	−0.017	yes
Married	0.024	−1.056	1.109	No

**R-squared**	**F value**	**p** **value**		
0.199	57.690	< 0.001		

*Analysis of Total Effect, Direct Effect, and Mediating Effect*				
PW ⟶ PG fit(a)	0.237	0.180	0.294	yes
PG fit ⟶ quiet quitting (b)	−0.371	−0.528	−0.217	yes
Indirect effect (a × b)	−0.088	−0.136	−0.046	yes
Direct effect (c′)	−0.218	−0.299	−0.139	yes
Total effect (c)	−0.305	−0.358	−0.252	yes

*Note:* PG fit = person‐group fit, PW = psychological well‐being.

### 3.4. Moderated Mediation Model Results

We tested the proposed moderated mediation model using Hayes’ PROCESS macro (Model 59), with Generation specified as a moderator of the component paths in the model. Results indicated that Generation moderated some individual associations within the model. For the association between PWB and PG fit (a path), the interaction effect for Generation X was significant (*β* = −0.225, *t* = −2.706, *p* = 0.007, 95% CI [−0.389, −0.062]), indicating that the positive association between PWB and PG fit was weaker among Generation X nurses compared with Generation Z nurses. No significant moderation effect was observed for Generation Y on this path.

For the direct effect of PWB on QQ (c′ path), significant moderation effects were observed for Generation Y (*β* = −0.141, *p* = 0.048) and Generation X (*β* = −0.201, *p* = 0.016). These interaction effects indicated that the negative association between PWB and QQ differed across generations, with stronger associations observed among Generation Y and Generation X relative to Generation Z. However, the magnitude of these effects was relatively small. For the b path (PG fit ⟶ QQ), a significant moderation effect was found for Generation Y (*β* = 0.273, *t* = 3.832, *p* < 0.001, 95% CI [0.133, 0.413]), indicating that the negative association between PG fit and QQ was weaker among Generation Y compared with Generation Z. No significant moderation effect was observed for Generation X on this path.

To assess moderated mediation, we examined the index of moderated mediation. The indirect effects did not significantly differ between Generation Z and either Generation Y (Index = 0.120, BootSE = 0.055, 95% CI [−0.006, 0.209]) or Generation X (Index = 0.123, BootSE = 0.056, 95% CI [−0.018, 0.207]), as both confidence intervals included zero. Thus, there was no evidence that the indirect effect of PWB on QQ through PG fit differed significantly across generations.

Because the indices of moderated mediation were not statistically significant, conditional indirect effects are presented for descriptive purposes only. The indirect effect of PWB on QQ through PG fit was statistically significant for Generation Z (Effect = −0.183, 95% CI [−0.244, −0.116]) and Generation X (Effect = −0.061, 95% CI [−0.177, −0.005]), but not for Generation Y (Effect = −0.063, 95% CI [−0.166, 0.006]). Although the indirect effect appeared numerically larger among Generation Z nurses, the nonsignificant indices of moderated mediation indicate that these observed differences should not be interpreted as evidence of statistically significant differences between cohorts.

Overall, although the conditional indirect effects were generally negative, the nonsignificant indices of moderated mediation indicate that there is insufficient evidence to conclude that the indirect effect differs across generations (Table 5). Therefore, any apparent cohort differences should be regarded as exploratory rather than confirmatory.

**TABLE 5:** Moderated mediation analysis when using generation as a moderator (*N* = 977).

	Outcome variable: PG_fit			
	*β*	SE	*T*	*p*
*Mediator Variable Model*				
PW	0.427	0.048	8.881	< 0.001
GenY	0.052	0.101	0.52	0.603
GenX	−0.047	0.188	−0.253	0.801
PW ∗ GenY	−0.025	0.068	−0.361	0.718
PW ∗ GenX	−0.225	0.083	−2.706	0.007

	**Outcome Variable: Quiet Quitting**			
	***β***	**SE**	***t***	**p**

*Dependent Variable Model*				
PWB	−0.132	0.051	−2.583	0.010
PG fit	−0.43	0.054	−7.951	< 0.001
GenY	0.232	0.095	2.432	0.015
GenX	0.065	0.178	0.368	0.713
PWB ∗ GenY	−0.141	0.071	−1.982	0.048
PWB ∗ GenX	−0.201	0.083	−2.418	0.016
PG fit ∗ GenY	0.273	0.071	3.832	< 0.001
PG fit ∗ GenX	0.128	0.086	1.496	0.135

**Generation**	**Indirect Effect**	**BootSE**	**95% CI (LL, UL)**	

*Conditional Indirect Effects*				
Gen Z	−0.183	0.033	[–0.244, −0.116]	
Gen Y	−0.063	0.044	[–0.166, 0.006]	
Gen X	−0.061	0.045	[–0.177, −0.005]	

**Comparison**	**Index**	**BootSE**	**95% CI (LL, UL)**	

*Index of Moderated Mediation (Generation Comparisons)*				
Gen Y vs. Gen Z	0.120	0.055	[–0.006, 0.209]	
Gen X vs. Gen Z	0.123	0.056	[–0.018, 0.207]	

*Note:* PWB = psychological well‐being, PG fit = person‐group fit, GenY = Generation Y, GenX = Generation X.

To illustrate the moderating effect of generation on the relationship between PWB and PG fit, conditional effects were estimated at the mean and ±1 SD of PW for each generation. PW was positively associated with PG fit across all generations (*p* < 0.01). The association was strongest for Generation Z (B = 0.427), followed by Generation Y (B = 0.402), and weakest for Generation X (B = 0.201). Although the pattern suggests some variation across cohorts, the differences in effect size were modest. Consistent with this pattern, the PW × Generation X interaction was significant (*p* = 0.007), indicating that the positive association between PW and PG fit was weaker among Generation X nurses.

PW was also associated with lower levels of QQ across all generations (*p* < 0.05). The relationship remained significant for Generation Y (*p* = 0.048) and Generation X (*p* = 0.016), although the effects were weaker than those observed for Generation Z. Overall, the differences across cohorts were relatively small, suggesting only modest variation in the relationship between PW and QQ by generation.

PG fit was negatively associated with QQ across all generations (*p* < 0.01). The relationship was strongest for Generation Z (B = −0.430, *p* < 0.001), followed by Generation X (B = −0.302, *p* < 0.001), and weakest for Generation Y (B = −0.157, *p* = 0.001). The interaction term for Generation Y was significant (*p* < 0.001), indicating that generation moderated the association between PG fit and QQ, whereas the interaction term for Generation X was not significant. These findings suggest that PG fit consistently predicts lower QQ across cohorts, although the strength of this relationship varies modestly by generation.

**FIGURE 2:**
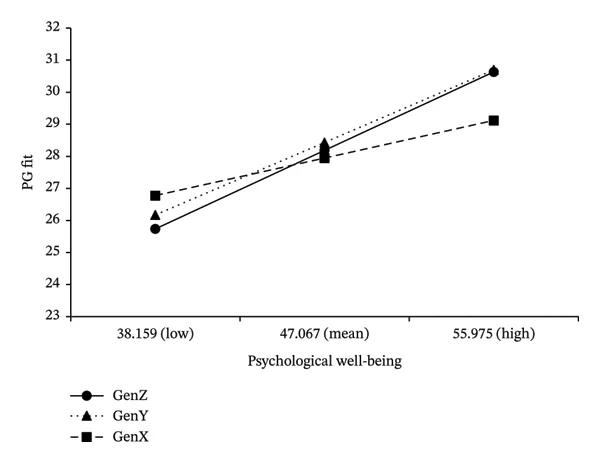
Moderating effect of generation on the relationship between psychological well‐being and person‐group fit. The positive association between psychological well‐being and person‐group fit is strongest for Gen Z and weakest for Gen X. Note. The x‐axis displays raw psychological well‐being scores. Low, mean, and high represent the raw score values corresponding to M − 1 SD, M, and M + 1 SD, respectively. The original psychological well‐being scale ranges from 14 to 70.

**FIGURE 3:**
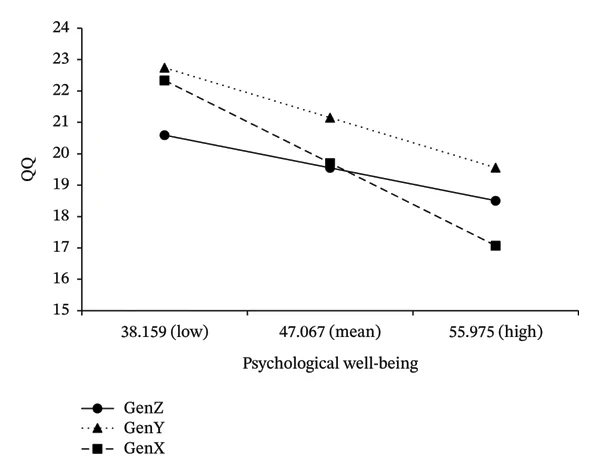
Moderating effect of generation on the relationship between psychological well‐being and QQ. The negative association between psychological well‐being and QQ is stronger among Gen Z than among Gen Y and Gen X. Note. The x‐axis displays raw psychological well‐being scores. Low, mean, and high represent the raw score values corresponding to M − 1 SD, M, and M + 1 SD, respectively. The original psychological well‐being scale ranges from 14 to 70.

**FIGURE 4:**
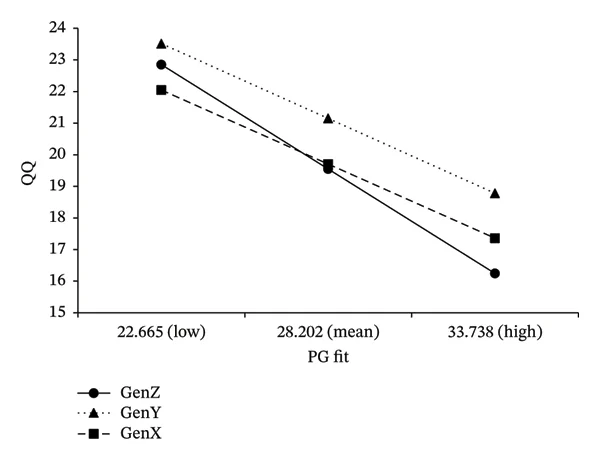
Moderating effect of generation on the relationship between person‐group (PG) fit and QQ. The negative association between PG fit and QQ appears strongest among Gen Z and weakest among Gen Y. Note. The X‐axis displays raw person‐group (PG) fit scores. Low, mean, and high represent the raw score values corresponding to M − 1 SD, M, and M + 1 SD, respectively. The original PG fit scale ranges from 6 to 42.

Figures 2–4 illustrate the conditional effects for the individual model pathways. Although some pathway‐specific moderation effects were observed, the nonsignificant indices of moderated mediation indicate that these effects did not result in statistically significant differences in the overall indirect effect across generations. Therefore, differences in individual pathway estimates across cohorts should be interpreted as exploratory and not as evidence of differences in the overall indirect effect.

### 3.5. Additional Sensitivity Analyses

To assess the robustness of the findings to alternative covariate specifications, we conducted sensitivity analyses by adding age and clinical unit type to the primary model. Because age was highly correlated with tenure, which was retained in the primary model based on its theoretical relevance to workplace perceptions and organizational exposure, these analyses were treated as robustness checks rather than alternative primary models. The results were substantively consistent with those of the primary analyses, with no meaningful changes in the estimated coefficients, significance levels, or indirect effects (see Supporting Tables X1–X4). Shift schedule could not be examined because this variable was not collected in the present study.

## 4. Discussion

### 4.1. PG Fit Mediates the Relationship Between PWB and QQ

The results indicate that PWB reduces QQ both directly and indirectly via enhanced PG fit. Although both effects were statistically significant, the indirect effect was relatively modest in magnitude, suggesting that PG fit represents one contributing pathway linking PWB and QQ rather than a dominant explanatory mechanism. Nurses with higher PWB may be less inclined to disengage and more likely to experience alignment and belonging within their teams, thereby reducing withdrawal behaviors. This finding is consistent with PG fit theory, which posits that congruence between individual values and group norms promotes favorable work outcomes [16].

Beyond PG fit theory, SET and conservation of resources (COR) theory also help explain this relationship [45–47]. Nurses with greater PWB possess stronger emotional and cognitive resources, which may facilitate interpersonal engagement, helping behaviors, and trust‐building within teams. Positive affect may also spread through emotional contagion, reinforcing collaborative norms and perceived belonging. Over time, these resource‐ and affect‐based processes may strengthen alignment with group values, particularly in supportive and cohesive team environments. In turn, stronger PG fit may contribute to lower QQ by enhancing social integration and engagement within the workplace; however, the observed effect suggests that PG fit is one of multiple factors influencing withdrawal‐related behaviors [48, 49].

These findings extend prior research by identifying PG fit as a potential relational mechanism linking PWB to QQ among nurses. Whereas previous studies have primarily emphasized structural drivers such as workload, burnout, and staffing shortages [1, 50], the present study highlights psychological and relational processes within work teams. Consistent with PG fit theory [16] and prior evidence linking well‐being to sustained engagement and professional commitment [51, 52], alignment with work groups appears to contribute to lower levels of behavioral withdrawal. Importantly, these findings support the conceptualization of QQ as a form of behavioral withdrawal characterized by reduced discretionary effort rather than full psychological detachment or explicit turnover intention.

Although related to reduced OCB and covert withdrawal intentions, QQ may represent a more gradual and relationally embedded response to perceived imbalances in workplace exchange relationships, particularly in highly interdependent nursing environments.

The relevance of PG fit may vary across cultural contexts. In collectivist cultures such as Taiwan, where interpersonal interdependence, group harmony, and hierarchical workplace relationships are emphasized, alignment with work groups may be particularly important in shaping work attitudes and disengagement behaviors. This suggests that PG fit may represent a culturally contingent mechanism in explaining QQ, although this interpretation remains exploratory. Future cross‐cultural research is needed to examine whether the magnitude of the PG fit pathway differs across cultural settings and more individualistic contexts.

From a practical perspective, reducing QQ in nursing requires attention to both individual and collective resources. Organizational practices such as empathetic supervision, opportunities for peer connection, equitable workload distribution, and recognition systems may simultaneously enhance PWB and strengthen PG fit. Consistent with PG fit theory, fostering value–norm congruence can promote positive work behaviors [53]. However, given the modest magnitude of the observed effects, interventions targeting PWB and group fit should be viewed as incremental strategies that may contribute to reducing QQ rather than as standalone solutions. By supporting nurses’ psychological resources while cultivating cohesive team environments, healthcare organizations may more effectively sustain engagement and mitigate disengagement in clinical settings [9], although the effects are likely to be incremental rather than large in magnitude.

### 4.2. Moderated Mediation

The moderated mediation analysis examined whether generational cohort influenced the indirect effect of PWB on QQ via PG fit. Conditional indirect effects were descriptively strongest for Generation Z, weaker for Generation Y, and smaller for Generation X. Although the conditional indirect effects for Generation Z and Generation X reached statistical significance, the overall index of moderated mediation was not significant, indicating that these apparent cohort differences should be interpreted cautiously.

Although the moderated mediation hypothesis was not supported, the pattern of conditional effects may provide descriptive insight into potential cohort‐related variation. Among Generation Z nurses, the indirect effect through PG fit was more pronounced, suggesting that PWB may be more closely linked to disengagement via perceived group alignment in this cohort. This pattern is broadly consistent with prior research indicating that younger employees tend to place greater emphasis on interpersonal support, social belonging, and value congruence in the workplace [23].

For Generation Y and Generation X nurses, the indirect effects were smaller in magnitude, indicating weaker evidence for a mediating role of PG fit in these cohorts. These smaller effects may reflect differences in work priorities or career circumstances, but they cannot be interpreted as evidence of distinct psychological mechanisms across generations.

From the perspective of Career Stage Theory [54], the weaker conditional indirect effect observed among Generation Y nurses may reflect differences in work priorities associated with the mid‐career stage. Mid‐career nurses often balance expanding professional responsibilities with family commitments and may therefore place greater emphasis on work‐family balance, career development opportunities, and job security than on achieving value congruence with their immediate work group. Consequently, although PWB remains important, its influence on QQ may be less likely to operate through perceptions of PG fit in this cohort.

This interpretation is also broadly consistent with Socioemotional Selectivity Theory [55], which suggests that work‐related goals vary as individuals’ future time perspectives change across the life course. Compared with nurses at later career stages, mid‐career nurses may be relatively more oriented toward instrumental goals, such as career advancement and role development, whereas interpersonal fit within the immediate work group may play a comparatively smaller role in shaping the relationship between PWB and QQ. However, because the index of moderated mediation was not statistically significant, this interpretation should be viewed as a theoretically informed possibility rather than evidence of distinct generational mechanisms.

More importantly, the nonsignificant moderated mediation has theoretical implications beyond the absence of a statistical interaction. It suggests that the psychological process linking PWB to QQ through PG fit may be more broadly applicable across generational cohorts than accounts portraying QQ as a distinctly generational phenomenon would suggest. Specifically, the lack of significant differences in the overall indirect effect suggests that the role of PG fit in this process does not systematically vary across generations. This finding provides an important theoretical qualification to generational accounts of QQ by suggesting that generation may be less central to explaining the underlying psychological process than commonly assumed. Future longitudinal and cross‐cohort research should examine whether career stage, workplace priorities, and contextual factors, rather than generational membership per se, better explain variation in this process.

### 4.3. Implications for Practice

This study provides practical insights into how nursing managers may reduce QQ by strengthening PWB and relational alignment within multigenerational teams. Rather than treating nurses as homogeneous groups, managers should consider differences in workplace expectations, career stages, and motivational priorities when designing engagement strategies [56, 57].

When nurses perceive organizational support and concern for their well‐being, they are more likely to experience stronger PG fit, which in turn may reduce disengagement behaviors through enhanced alignment with team values and interpersonal norms [58, 59].

From a practical perspective, these findings suggest that interventions aimed at reducing QQ should strengthen both PWB and relational alignment within teams. Strategies such as structured onboarding, clear communication of unit values, and team socialization activities may facilitate early integration into team norms and enhance PG fit. In addition, formal mentorship programs that connect less experienced nurses with senior staff may support interpersonal trust and adaptation to collaborative clinical workflows [60].

To further address the needs of a multigenerational nursing workforce, healthcare managers should consider cohort‐sensitive strategies that reflect differences in career stage, workplace expectations, and motivational priorities. For Generation Z nurses, who are often in the early stage of professional socialization, structured onboarding programs, explicit communication of team values, and opportunities for reverse mentoring may be particularly beneficial. These approaches may facilitate adjustment to clinical environments, strengthen a sense of belonging, and enhance perceptions of PG fit during the transition into professional practice.

For Generation Y nurses, who are commonly situated in the mid‐career stage, engagement strategies may need to recognize the competing demands of professional advancement and personal responsibilities. Flexible scheduling options, transparent career development pathways, and constructive performance feedback may help support work‐family balance, career sustainability, and continued engagement [61]. By addressing these priorities, organizations may strengthen PWB and promote greater alignment between individual career goals and team values.

For Generation X nurses, who often have accumulated substantial clinical experience and professional expertise, strategies that reinforce autonomy and professional contribution may be especially important [62, 63]. Participative decision‐making opportunities, clinical mentorship roles, and meaningful professional recognition may strengthen their sense of value within the team and reinforce commitment to shared team goals.

Beyond cohort‐sensitive approaches, healthcare organizations should also foster intergenerational collaboration to facilitate knowledge sharing, mutual understanding, and collective team identity across generations. Such approaches may help create conditions conducive to PG fit by promoting shared values and cooperative relationships within diverse nursing teams [60]. However, implementing these strategies may be challenging in practice given persistent nursing shortages, heavy workloads, staffing pressures, and limited managerial time and resources. A phased implementation approach may therefore be more feasible, beginning with low‐cost, high‐impact strategies that can be embedded in existing management routines. For example, managers could incorporate brief team‐based value communication into regular meetings and establish structured mentorship or peer‐support arrangements that require limited additional resources. More resource‐intensive initiatives, such as flexible scheduling and formal career development programs, could then be introduced progressively as organizational capacity permits. This approach may enhance the feasibility and sustainability of efforts to strengthen PWB and PG fit without adding substantial demands to already constrained nursing environments. Overall, the findings support flexible leadership approaches that attend to PWB, cohort‐sensitive needs, and team‐based alignment and communication [64]. By integrating feasible individual‐ and team‐focused interventions, healthcare managers may better address the multiple pathways associated with QQ in a diverse nursing workforce [23, 65].

### 4.4. Limitations and Future Research

A key strength of this study is its three‐wave design, which helps reduce common method bias and allows a more robust examination of the proposed mediation and moderation processes. However, the findings remain noncausal and should be interpreted with caution. Future longitudinal research is needed to further clarify the temporal dynamics among PWB, PG fit, and QQ. The one‐to two‐month intervals between waves were selected based on the expected short‐to medium‐term fluctuations of work‐related psychological states and behaviors; however, these relatively short intervals may primarily capture proximal changes rather than longer‐term developmental processes. For example, cumulative effects associated with prolonged occupational stress, burnout, evolving professional identity, or changes in organizational attachment may require longer observation periods to emerge. Therefore, the current findings may underestimate delayed or cumulative relationships among PWB, PG fit, and QQ. Future research should consider longer follow‐up periods and employ cross‐lagged panel designs or other extended longitudinal approaches to examine reciprocal and cumulative effects among these constructs.

Another limitation concerns the use of self‐reported measures, which may still introduce common method variance and response biases [66]. Future studies could strengthen methodological rigor by incorporating multisource data (e.g., supervisor or peer ratings) and applying advanced statistical controls such as marker variables or latent method factor approaches [66, 67]. In addition, the present study did not collect information on shift schedule or more detailed indicators of nursing workload (e.g., nurse‐to‐patient ratios, patient acuity, or skill mix), which may influence PWB, PG fit, and QQ. Future research should incorporate these contextual factors to provide a more comprehensive understanding of nurses’ work experiences. Qualitative or mixed‐method designs may also provide deeper insight into the subjective experiences and contextual drivers of QQ. Future research is also encouraged to examine whether the proposed relationships hold across alternative and potentially longer time lags.

The sample was drawn from a single medical center in Taiwan, which may limit generalizability to other cultural and organizational contexts. Taiwanese healthcare organizations are characterized by relatively high collectivism and power distance [19, 68]. In such settings, employees may place greater emphasis on group harmony, interpersonal obligations, and team belonging, which may heighten the salience of PG fit [69, 70]. At the same time, high power distance may discourage overt forms of resistance or withdrawal behaviors [71]. Accordingly, QQ in such contexts may manifest less through observable behavioral withdrawal and more through subtle forms of psychological disengagement, reduced enthusiasm, passive compliance, or diminished discretionary investment that are not readily observable.

Importantly, this raises a potential cultural measurement limitation: the present study operationalizes QQ through self‐reported indicators of reduced discretionary effort, work disengagement, and diminished psychological investment. Although these indicators capture multiple manifestations of QQ, some culturally constrained or low‐visibility forms of disengagement may remain difficult to detect, particularly when employees avoid explicit expressions of withdrawal in high power‐distance contexts. Although the adapted QQ scale demonstrated acceptable psychometric performance in the present nursing sample, QQ remains an evolving construct in healthcare research. Additional validation across diverse clinical and cultural settings is needed to further establish the generalizability and cultural sensitivity of the measure.

Generational differences were examined using birth cohorts as a proxy for generational identity. While this approach is widely used in organizational and nursing research, it represents a pragmatic rather than definitive operationalization of generational differences. However, it may not fully capture within‐cohort heterogeneity and may overlap with age‐, career stage‐, and period‐related effects. Therefore, the findings should be interpreted as cohort‐based associations rather than causal generational effects. Future research could incorporate direct measures of generational values or identity and apply age–period–cohort designs to better disentangle these effects and provide a more nuanced understanding of workforce differences in healthcare settings [72, 73].

Finally, future studies may examine additional contextual moderators, such as leadership style or organizational culture, which may shape the relationship between PG fit, PWB, and QQ. Comparative studies across different healthcare professions and organizational types would also enhance external validity and clarify the generalizability of the proposed mechanisms.

## 5. Conclusion

This study demonstrates that PWB reduces QQ through enhanced PG fit, with the strength of this pathway differing across generations. For Generation Z, PG fit was the primary mechanism linking well‐being to engagement, whereas for older cohorts the PG fit–QQ association was stronger. These findings highlight the need for generationally responsive strategies that foster well‐being, strengthen value congruence within teams, and address the distinct drivers of disengagement across age groups. In the postpandemic workplace, creating supportive environments that integrate individual psychological resources with collective cohesion may help mitigate QQ and support a resilient nursing workforce.

## Funding

This study was supported by the National Science and Technology Council, NSTC 113‐2410‐H‐110‐015‐SS2.

## Conflicts of Interest

The authors declare no conflicts of interest.

## Endnotes

^1^The item was excluded during content validation because the expert panel did not consider it sufficiently representative of the construct in the nursing context.

## Supporting Information

Additional supporting information can be found online in the Supporting Information section.

## Supporting information


**Supporting Information 1** Supporting 1. Appendix A. Nomological network of quiet quitting and related employee work behavior constructs.

**Supporting Information 2** Supporting 2. Appendix B. Items of the quiet quitting scale.

**Supporting Information 3** Supporting 3. Table S1. Confirmatory factor analysis (CFA) results of the quiet quitting scale.

**Supporting Information 4** Supporting 4. Table S2. Measurement invariance testing across generational groups.

**Supporting Information 5** Table X1. Sensitivity analysis of the mediation model with age included as an additional control variable.

**Supporting Information 6** Table X2. Sensitivity analysis of the moderated mediation model with age included as an additional control variable.

**Supporting Information 7** Table X3. Sensitivity analysis of the mediation model with age and unit type included as additional control variables.

**Supporting Information 8** Table X4. Sensitivity analysis of the moderated mediation model with age and unit type included as additional control variables.

## Data Availability

The datasets generated and/or analyzed during the current study are not publicly available due to ethical and confidentiality restrictions involving human participants. They may be available from the corresponding author, Dr. Shu‐Chuan Jennifer Yeh, upon reasonable request and subject to appropriate ethical review and approval where required.
