# Supplementary material for: Quiet Quitting in Nursing: The Role of Psychological Well‐Being, Person‐Group Fit, and Generational Differences
Source: J Nurs Manag. 2026 Sep 18;2026:2836032. doi: 10.1155/jonm/2836032 (PMC13589159; doi:10.1155/jonm/2836032)
Supplement: Supplementary file 1 [file JONM-2026-2836032-s001.docx]

Table S2. Measurement invariance testing across generational groups

| **Model** | **Model description** | **χ^2^** | **df** | **CFI** | **RMSEA** | **Δχ^2^** | **Δdf** | **sig.** | **ΔCFI** | **ΔRMSEA** |
| --- | --- | --- | --- | --- | --- | --- | --- | --- | --- | --- |
| 1 | Configural invariance (baseline) | 257.143 | 42 | 0.929 | 0.073 | - | - | - | - | - |
| 2 | Metric invariance | 270.036 | 54 | 0.926 | 0.066 | 12.893 | 12 | 0.37687 | 0.003 | 0.007 |
