# Supplementary material for: Quiet Quitting in Nursing: The Role of Psychological Well‐Being, Person‐Group Fit, and Generational Differences
Source: J Nurs Manag. 2026 Sep 18;2026:2836032. doi: 10.1155/jonm/2836032 (PMC13589159; doi:10.1155/jonm/2836032)
Supplement: Supplementary file 2 [file JONM-2026-2836032-s002.docx]

Table S1. Confirmatory Factor Analysis (CFA) Results of the Quiet Quitting Scale

|  | All | | | Gen X | | |
| --- | --- | --- | --- | --- | --- | --- |
|  | **factor loading** | **CR** | **AVE** | **factor loading** | **CR** | **AVE** |
| Item 1 | 0.62 | 0.862 | 0.582 | 0.61 | 0.881 | 0.625 |
| Item 2 | 0.72 |  |  | 0.77 |  |  |
| Item 3 | 0.79 |  |  | 0.79 |  |  |
| Item 4 | 0.86 |  |  | 0.91 |  |  |
| Item 5 | 0.73 |  |  | 0.73 |  |  |
| Item 6 | 0.62 |  |  | 0.72 |  |  |
| Item 7 | 0.63 |  |  | 0.64 |  |  |
| χ²(df) | 191.959(14) |  |  | 52.36(14) |  |  |
| CFI | 0.938 |  |  | 0.944 |  |  |
| TLI | 0.906 |  |  | 0.917 |  |  |
| RMSEA | 0.082 |  |  | 0.079 |  |  |
| SRMR | 0.0477 |  |  | 0.0455 |  |  |
|  | **Gen Y** | | | **Gen Z** | | |
|  | **factor loading** | **CR** | **AVE** | **factor loading** | **CR** | **AVE** |
| Item 1 | 0.62 | 0.835 | 0.533 | 0.63 | 0.884 | 0.63 |
| Item 2 | 0.66 |  |  | 0.73 |  |  |
| Item 3 | 0.78 |  |  | 0.76 |  |  |
| Item 4 | 0.84 |  |  | 0.84 |  |  |
| Item 5 | 0.69 |  |  | 0.78 |  |  |
| Item 6 | 0.65 |  |  | 0.84 |  |  |
| Item 7 | 0.67 |  |  | 0.63 |  |  |
| χ²(df) | 69.473(14) |  |  | 135.294(14) |  |  |
| CFI | 0.945 |  |  | 0.938 |  |  |
| TLI | 0.918 |  |  | 0.908 |  |  |
| RMSEA | 0.088 |  |  | 0.076 |  |  |
| SRMR | 0.0456 |  |  | 0.0478 |  |  |
