# Supplementary material for: Quiet Quitting in Nursing: The Role of Psychological Well‐Being, Person‐Group Fit, and Generational Differences
Source: J Nurs Manag. 2026 Sep 18;2026:2836032. doi: 10.1155/jonm/2836032 (PMC13589159; doi:10.1155/jonm/2836032)
Supplement: Supplementary file 3 [file JONM-2026-2836032-s003.docx]

**Appendix B: Items of Quiet quitting**

1. I often arrive late and leave early from work

2. I do only the minimum required to fulfill my job responsibilities.

3. I feel there is a lack of opportunities to learn and grow in my organization

4. I feel there is a lack of meaningfulness at work

5. I feel I have a lack of interest in attending meetings

6. I feel there is a lack of passion and enthusiasm in me to work above and beyond

7. I feel that my supervisor does not show care or concern for me.

Note: Items were retained from the adapted quiet quitting scale developed by Anand et al. (2023). Items reflect behavioral withdrawal tendencies and psychological disengagement dimensions associated with quiet quitting.
