# Supplementary material for: Quiet Quitting in Nursing: The Role of Psychological Well‐Being, Person‐Group Fit, and Generational Differences
Source: J Nurs Manag. 2026 Sep 18;2026:2836032. doi: 10.1155/jonm/2836032 (PMC13589159; doi:10.1155/jonm/2836032)
Supplement: Supplementary file 4 [file JONM-2026-2836032-s004.docx]

**Appendix A. Nomological Network of Quiet Quitting and Related Employee Work Behavior Constructs**

| **Construct** | **Primary focus** | **Employee status** | **In-role performance** | **Discretionary extra-role contribution** | **Psychological meaning** |
| --- | --- | --- | --- | --- | --- |
| Quiet quitting | Restricting effort to contractual requirements | Yes | Yes | Low | Deliberate reduction of discretionary effort while maintaining formal role performance |
| Work engagement | Positive work involvement | Yes | Yes | High | High vigor, dedication, and absorption |
| Organizational Citizenship Behavior | Voluntary contribution | Yes | Yes | High | Willingness to exceed formal role expectations |
| Work withdrawal | Behavioral withdrawal | Sometimes | Reduced | Reduced | Psychological or behavioral disengagement |
| Turnover intention | Intention to leave | Currently yes | Usually yes | Variable | Cognitive intention to leave the organization |
