# Supplementary material for: Quiet Quitting in Nursing: The Role of Psychological Well‐Being, Person‐Group Fit, and Generational Differences
Source: J Nurs Manag. 2026 Sep 18;2026:2836032. doi: 10.1155/jonm/2836032 (PMC13589159; doi:10.1155/jonm/2836032)
Supplement: Supplementary file 5 [file JONM-2026-2836032-s005.docx]

**Table X1. The Mediation model of Person-Group Fit**

| **Variables** | **coefficient** | **Boot LLCI** | **Boot ULCI** | **Significance** |
| --- | --- | --- | --- | --- |
| **Outcome variable: PG Fit** | | | | |
| Constant | 18.639 | 15.733 | 21.545 | yes |
| PW | 0.239 | 0.201 | 0.276 | yes |
| Tenure | 0.057 | -0.041 | 0.155 | No |
| Married | 0.694 | -0.110 | 1.498 | No |
| Age | -0.074 | -0.171 | 0.023 | No |
| **R-squared** | **F value** | **P value** |  |  |
| 0.153 | 41.422 | <0.001 |  |  |
| **Outcome variable: Quiet Quitting** | | | | |
| Constant | 43.084 | 38.998 | 47.17 | yes |
| PW | –0.188 | -0.24 | -0.135 | yes |
| PG fit | -0.473 | -0.557 | -0.389 | yes |
| Tenure | -0.069 | -0.196 | 0.059 | yes |
| Married | 0.213 | -0.832 | 1.258 | No |
| Age | 0.002 | -0.125 | 0.128 | No |
| **R-squared** | **F value** | **P value** |  |  |
| 0.239 | 57.438 | <0.001 |  |  |
| **Analysis of total effect, direct effect, and mediating effect** | | | | |
| **PW → PG fit（a）** | 0.239 | 0.201 | 0.276 | yes |
| **PG fit → quiet quitting (b)** | –0.473 | -0.557 | -0.389 | yes |
| **Indirect effect**  **(a × b）** | –0.113 | -0.162 | -0.072 | yes |
| **Direct effect（c′）** | -0.188 | -0.24 | -0.135 | yes |
| **Total effect (c）** | -0.301 | -0.352 | -0.249 | yes |

Notes: PG fit = person-group fit; PW = psychological well-being; Tenure, married, and age are included as controls
