# Supplementary material for: Quiet Quitting in Nursing: The Role of Psychological Well‐Being, Person‐Group Fit, and Generational Differences
Source: J Nurs Manag. 2026 Sep 18;2026:2836032. doi: 10.1155/jonm/2836032 (PMC13589159; doi:10.1155/jonm/2836032)
Supplement: Supplementary file 6 [file JONM-2026-2836032-s006.docx]

**Table X3. The Mediation model of Person-Group Fit**

| **Variables** | **coefficient** | **Boot LLCI** | **Boot ULCI** | **Significance** |
| --- | --- | --- | --- | --- |
| **Outcome variable: PG Fit** | | | | |
| Constant | 18.407 | 15.339 | 21.474 | yes |
| PW | 0.238 | 0.201 | 0.276 | yes |
| Tenure | 0.055 | -0.044 | 0.153 | No |
| Married | 0.716 | -0.094 | 1.525 | No |
| Age | -0.07 | -0.169 | 0.028 | No |
| Unit type | 0.006 | -0.018 | 0.029 | No |
| **R-squared** | **F value** | **P value** |  |  |
| 0.154 | 33.153 | <0.001 |  |  |
| **Outcome variable: Quiet Quitting** | | | | |
| Constant | 43.498 | 39.226 | 47.77 | yes |
| PW | -0.187 | -0.24 | -0.135 | yes |
| PG fit | -0.473 | -0.557 | -0.388 | yes |
| Tenure | -0.065 | -0.193 | 0.063 | No |
| Married | 0.172 | -0.88 | 1.225 | No |
| Age | -0.005 | -0.133 | 0.123 | No |
| Unit type | -0.01 | -0.04 | 0.02 | No |
| **R-squared** | **F value** | **P value** |  |  |
| 0.239 | 47.906 | <0.001 |  |  |
| **Analysis of total effect, direct effect, and mediating effect** | | | | |
| **PW → PG fit（a）** | 0.238 | 0.201 | 0.276 | yes |
| **PG fit → quiet quitting (b)** | –0.473 | -0.557 | -0.388 | yes |
| **Indirect effect (a × b）** | –0.113 | -0.162 | -0.072 | yes |
| **Direct effect（c′）** | -0.187 | -0.24 | -0.135 | yes |
| **Total effect (c）** | -0.300 | -0.352 | -0.248 | yes |

Notes: PG fit = person-group fit; PW = psychological well-being; Tenure, married, age, and unit type as controls.
