# Supplementary material for: Quiet Quitting in Nursing: The Role of Psychological Well‐Being, Person‐Group Fit, and Generational Differences
Source: J Nurs Manag. 2026 Sep 18;2026:2836032. doi: 10.1155/jonm/2836032 (PMC13589159; doi:10.1155/jonm/2836032)
Supplement: Supplementary file 7 [file JONM-2026-2836032-s007.docx]

| **Table X4. Moderated mediation analysis when using generation as a moderator(N=977)** | | | | |
| --- | --- | --- | --- | --- |
| **Mediator variable model** | | | | |
|  | **Outcome variable: PG_fit** | | | |
|  | **β** | **SE** | **T** | **p** |
| PW | 0.435 | 0.048 | 8.987 | <0.001 |
| GenY | 0.187 | 0.125 | 1.504 | 0.133 |
| GenX | 0.229 | 0.246 | 0.929 | 0.353 |
| PW*GenY | -0.033 | 0.068 | -0.477 | 0.634 |
| PW*GenX | -0.237 | 0.084 | -2.822 | 0.005 |
| **Dependent variable model** | | | | |
|  | **Outcome variable: Quiet Quitting** | | | |
|  | **β** | **SE** | **t** | **p** |
| PW | -0.128 | 0.052 | -2.475 | 0.014 |
| PG_fit | -0.432 | 0.055 | -7.902 | <0.001 |
| GenY | 0.253 | 0.119 | 2.136 | 0.033 |
| GenX | 0.119 | 0.234 | 0.51 | 0.61 |
| PW*GenY | -0.144 | 0.072 | -2.019 | 0.044 |
| PW*GenX | -0.205 | 0.084 | -2.433 | 0.015 |
| PG_fit* GenY | 0.275 | 0.072 | 3.83 | <0.001 |
| PG_fit* GenX | 0.128 | 0.086 | 1.477 | 0.14 |
| **Conditional Indirect Effects** | | | | |
| **Generation** | **Indirect Effect** | **BootSE** | **95% CI (LL, UL)** | |
| Gen Z | –0.188 | 0.033 | [–0.251, –0.119] | |
| Gen Y | –0.063 | 0.044 | [–0.168, 0.007] | |
| Gen X | –0.06 | 0.044 | [–0.175, –0.005] | |
| **Index of Moderated Mediation (Generation Comparisons)** | | | | |
| **Comparison** | **Index** | **BootSE** | **95% CI (LL, UL)** | |
| Gen Y vs. Gen Z | 0.125 | 0.055 | [-0.002, 0.216] | |
| Gen X vs. Gen Z | 0.128 | 0.055 | [–0.005, 0.213] | |
| Notes: PW = psychological well-being; GenY = generation Y; GenX = generation X; PG_fit = Person group fit; Tenure, married, age, and unit type as controls. | | | | |
|  | | | | |
