# Supplementary material for: Quiet Quitting in Nursing: The Role of Psychological Well‐Being, Person‐Group Fit, and Generational Differences
Source: J Nurs Manag. 2026 Sep 18;2026:2836032. doi: 10.1155/jonm/2836032 (PMC13589159; doi:10.1155/jonm/2836032)
Supplement: Supplementary file 8 [file JONM-2026-2836032-s008.docx]

| **Table X2. Moderated mediation analysis when using generation as a moderator(N=977)** | | | | |
| --- | --- | --- | --- | --- |
| **Mediator variable model** | | | | |
|  | **Outcome variable: PG_fit** | | | |
|  | **β** | **SE** | **T** | **p** |
| PW | 0.435 | 0.048 | 8.985 | <0.001 |
| GenY | 0.178 | 0.124 | 1.44 | 0.15 |
| GenX | 0.23 | 0.246 | 0.936 | 0.35 |
| PW*GenY | -0.032 | 0.068 | -0.467 | 0.64 |
| PW*GenX | -0.234 | 0.084 | -2.798 | 0.005 |
| **Dependent variable model** | | | | |
|  | **Outcome variable: Quiet Quitting** | | | |
|  | **β** | **SE** | **t** | **p** |
| PW | -0.128 | 0.052 | -2.476 | 0.013 |
| PG_fit | -0.432 | 0.055 | -7.906 | <0.001 |
| GenY | 0.254 | 0.118 | 2.165 | 0.031 |
| GenX | 0.119 | 0.234 | 0.51 | 0.61 |
| PW*GenY | -0.144 | 0.071 | -2.02 | 0.044 |
| PW*GenX | -0.205 | 0.084 | -2.44 | 0.015 |
| PG_fit* GenY | 0.275 | 0.072 | 3.832 | <0.001 |
| PG_fit* GenX | 0.128 | 0.086 | 1.477 | 0.14 |
| **Conditional Indirect Effects** | | | | |
| **Generation** | **Indirect Effect** | **BootSE** | **95% CI (LL, UL)** | |
| Gen Z | –0.188 | 0.034 | [–0.251, –0.119] | |
| Gen Y | –0.063 | 0.045 | [–0.166, 0.008] | |
| Gen X | –0.061 | 0.045 | [–0.178, –0.004] | |
| **Index of Moderated Mediation (Generation Comparisons)** | | | | |
| **Comparison** | **Index** | **BootSE** | **95% CI (LL, UL)** | |
| Gen Y vs. Gen Z | 0.125 | 0.055 | [0.002, 0.221] | |
| Gen X vs. Gen Z | 0.127 | 0.056 | [–0.007, 0.214] | |
| Notes: PW = psychological well-being; GenY = generation Y; GenX = generation X; PG_fit = Person group fit; Tenure, marital status, and age as controls. | | | | |
|  | | | | |
